# Aortic wall gadolinium enhancement in thoracic aortic aneurysm patients

**DOI:** 10.1186/1532-429X-15-S1-E78

**Published:** 2013-01-30

**Authors:** Hadas Shiran, Bob S Hu, Michael V McConnell, David Liang

**Affiliations:** 1 Electrical Engineering, Stanford University, Palo Alto, CA, USA; 2 Cardiovascular Medicine, Stanford University, Palo Alto, CA, USA

## Background

Thoracic aortic aneurysm (TAA) disease is an often asymptomatic, insidious process that includes structural aortic changes, dilation, and eventually causes death. Aneurysm prevalence exceeds 4% and is the 15th leading cause of death in the US. TAA disease is associated with congenital and acquired defects in the aortic wall including elastin derangements, increased collagen, glycosaminolycan/proteoglycan accumulation, and loss of smooth muscle cells. We aim to investigate these changes non-invasively by studying the uptake of gadolinium (Gd) in the diseased aortic wall. The distribution and accumulation of Gd depends on tissue blood flow, the size of the interstitial space, and permeability of capillaries, and has potential to help characterize severity of TAA disease.

## Methods

**Figure 1:**
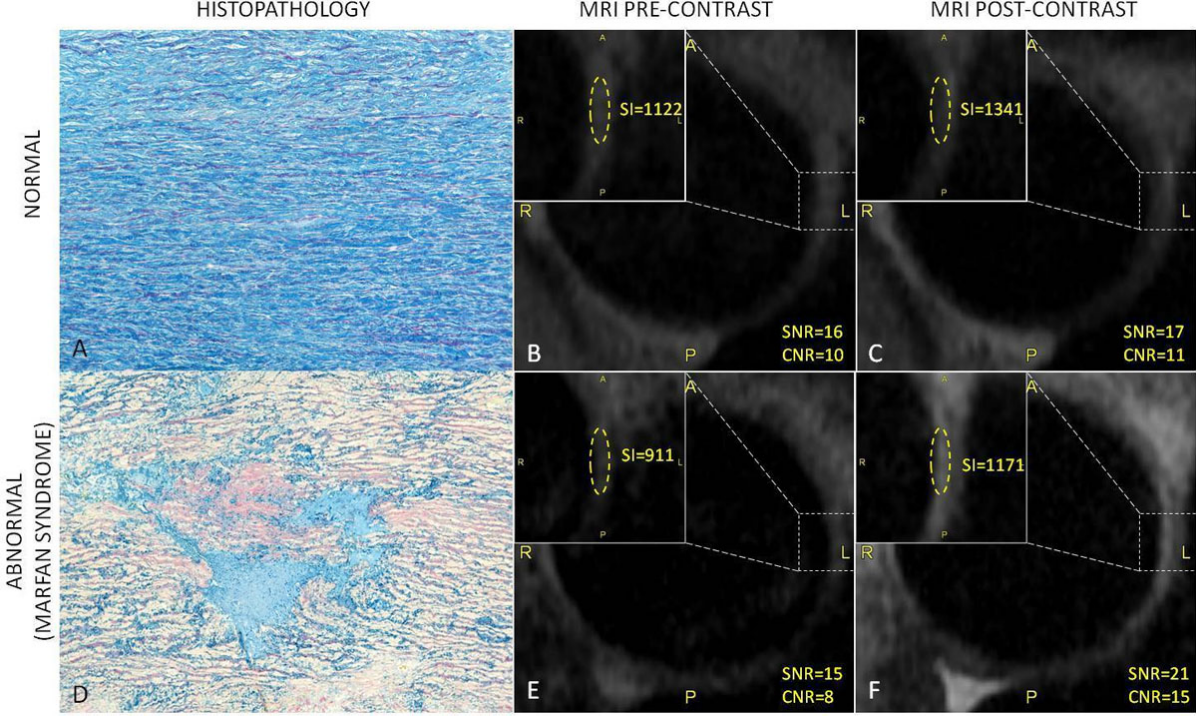
A) Normal colloidal iron stained aortic media at 10x magnification. B and C) Pre- and post-gadolinium proximal descending aortic wall in a control patient demonstrating minimal gadolinium uptake, change in SNR 7%, CNR 11% D) colloidal iron stain demonstrating severe medial myxoid degeneration in a patient with Marfan syndrome, 10x magnification. E and F) Pre- and post-gadolinium proximal descending aortic wall in a Marfan syndrome patient, demonstrating increased gadolinium uptake, change in SNR 43%, CNR 88%.

We recruited patients undergoing cardiac MR or thoracic MRA for clinical care at Stanford University Hospital on a 1.5T GE scanner. We imaged a cross-section of the thoracic aorta before and after Gd administration, using T1-weighted double inversion recovery in 9 patients, 5 with connective tissue disease (e.g. Marfan syndrome) and 4 controls. We used a small field of view, 18 cm, with matrix size 512 x 224, echo train length 16, slice thickness 8 mm, 2 R-R intervals, ECG-gating and breath-holding were used, with a 30 second acquisition. The post-Gd images were acquired 5-7 minutes after injection. For each segment of aorta imaged, SNR and CNR were calculated for the aortic wall before and after contrast (CNR was calculated vs. lumen using a 7-10 mm^2^ region-of interest (ROI) in the wall and 50-55 mm^2^ ROI in the lumen, as in Figure 1.

## Results

**Figure 2:**
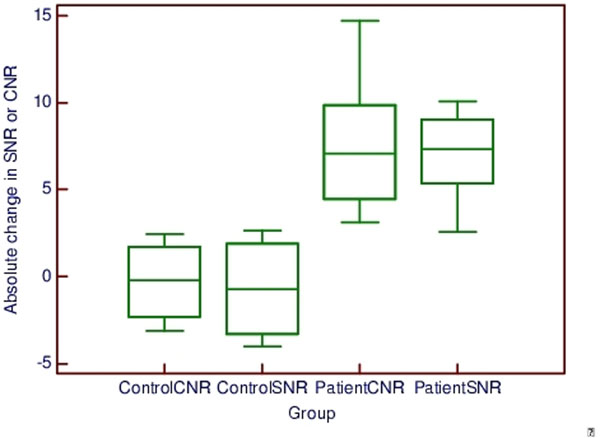
Comparison of SNR and CNR between patients and controls. Central box = 25-75 percentile, middle line = median. Whiskers = range.

We found significant post-Gadolinium enhancement of the aortic wall (7.0 +/- 2.9 absolute increase in SNR, 7.6 +/- 4.4 increase in CNR) in patients with aneurysms and connective tissue disease (Figure 2). By comparison, control patients had minimal evidence of Gd uptake (-0.7 +/- 3.1 decrease in SNR, -0.3 +/- 2.5 decrease in CNR). P < 0.05 for SNR and CNR comparisons in patients versus controls.

## Conclusions

Significant aortic wall Gd enhancement is seen in patients with connective tissue diseases and aneurysm formation and likely represents presence and severity of underlying tissue pathology.

## Funding

None.

